# Generating Synthetic Radiological Images with PySynthMRI: An Open-Source Cross-Platform Tool

**DOI:** 10.3390/tomography9050137

**Published:** 2023-09-11

**Authors:** Luca Peretti, Graziella Donatelli, Matteo Cencini, Paolo Cecchi, Guido Buonincontri, Mirco Cosottini, Michela Tosetti, Mauro Costagli

**Affiliations:** 1 Laboratory of Medical Physics and Magnetic Resonance, IRCCS Stella Maris, 56128 Pisa, Italy; luca.peretti@fsm.unipi.it (L.P.);; 2 Imago 7 Research Foundation, 56128 Pisa, Italy; 3 Department of Computer Science, University of Pisa, 56127 Pisa, Italy; 4 Department of Diagnostic and Interventional Radiology and Nuclear Medicine, Azienda Ospedaliero-Universitaria Pisana, 56124 Pisa, Italy; 5 Italian National Institute of Nuclear Physics (INFN), Section of Pisa, 56127 Pisa, Italy; 6 Department of Translational Research and New Technologies in Medicine and Surgery, University of Pisa, 56126 Pisa, Italy; 7 Department of Neuroscience, Rehabilitation, Ophthalmology, Genetics, Maternal and Child Sciences (DINOGMI), University of Genoa, 16132 Genoa, Italy

**Keywords:** magnetic resonance imaging, synthetic imaging, software tool

## Abstract

Synthetic MR Imaging allows for the reconstruction of different image contrasts from a single acquisition, reducing scan times. Commercial products that implement synthetic MRI are used in research. They rely on vendor-specific acquisitions and do not include the possibility of using custom multiparametric imaging techniques. We introduce PySynthMRI, an open-source tool with a user-friendly interface that uses a set of input images to generate synthetic images with diverse radiological contrasts by varying representative parameters of the desired target sequence, including the echo time, repetition time and inversion time(s). PySynthMRI is written in Python 3.6, and it can be executed under Linux, Windows, or MacOS as a python script or an executable. The tool is free and open source and is developed while taking into consideration the possibility of software customization by the end user. PySynthMRI generates synthetic images by calculating the pixelwise signal intensity as a function of a set of input images (e.g., T1 and T2 maps) and simulated scanner parameters chosen by the user via a graphical interface. The distribution provides a set of default synthetic contrasts, including T1w gradient echo, T2w spin echo, FLAIR and Double Inversion Recovery. The synthetic images can be exported in DICOM or NiFTI format. PySynthMRI allows for the fast synthetization of differently weighted MR images based on quantitative maps. Specialists can use the provided signal models to retrospectively generate contrasts and add custom ones. The modular architecture of the tool can be exploited to add new features without impacting the codebase.

## 1. Introduction

Magnetic Resonance Imaging (MRI) is a noninvasive modality that allows one to obtain images at a high spatial resolution with a wide range of radiologically meaningful structural soft tissue contrast and functional information. A complete MRI exam typically consists of the acquisition of several sequences, designed to obtain images with different contrasts, which depend on the differences in tissue properties, such as T1 and T2 relaxation times and proton density, and acquisition parameters, such as the times of repetition (TR), echo (TE) and inversion (TI). Each MRI sequence has a typical duration of several minutes and is designed to achieve only one specific tissue contrast. As a consequence, a complete MRI exam involves long acquisition times that can be challenging for children and uncollaborative patients. The recent development of methods for quantitative multiparametric mapping with a single time-efficient acquisition has paved the way for a new imaging approach, known as synthetic MRI, which aims to generate diverse contrast-weighted MR images from a set of tissue parameters [1,2,3,4]. This approach has been used and validated for clinical feasibility, and commercial products implementing synthetic MRI, such as SyMRI (SyntheticMR, Linköping, Sweden), are being used in research and clinical exams [5,6]. These products, however, rely on vendor-specific acquisitions and do not include the possibility of using custom implementations of multiparametric imaging techniques, such as MR Fingerprinting [7], MR-STAT [8], QALAS [9] and Quantitative Transient-state Imaging (QTI [10]). To the best of our knowledge, there is currently no open-source software available to perform synthetic MRI.

This article presents PySynthMRI, an open-source tool that uses a set of input images, such as quantitative maps of T1, T2 and proton density (PD), and allows the user to combine them to produce synthetic contrast-weighted images by varying simulated sequence parameters, such as TE, TR and TI(s). Pre-release versions of PySynthMRI have successfully been used to produce synthetic radiological images in the context of clinical research studies, recently presented in abstract form [11,12].

## 2. Method

**Figure 1:**
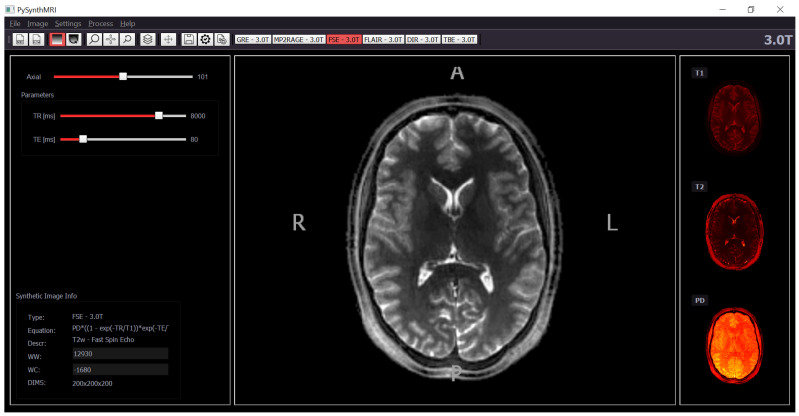
The graphical interface of PySynthMRI. On the left, the provided scanner parameter. In the middle, the synthesized FLAIR image. On the right, the input quantitative maps.

The PySynthMRI tool is written in Python 3.6, an open programming language available in all computing platforms. The tool makes use of the open source numpy and open-cv packages, chosen with the aim of maximizing the efficiency in manipulating image matrices and therefore of making the interaction with the graphical interface fluid for the user. The code is organized in a Model-View-Controller (MVC) pattern [13], and its modularity simplifies the tests and makes it possible to add new features without impacting the codebase. PySynthMRI has been developed in continuous collaboration with technicians and clinicians in order to obtain an easily usable interface, shown in Figure 1. PySynthMRI can be executed under Linux, Windows or MacOS as a python script or an executable. The minimum operating system requirements to run the software are Windows 7, Ubuntu 16.04, Red Hat Linux 6.6 or MacOS 10.11, depending on the system used. PySynthMRI can be executed on standard laptops with no special hardware requirements and is guaranteed to run on 2 GHz CPU and 4 GB RAM without a graphic card.

PyInstaller library has been used to bundle the tool and all its dependencies into a single executable package. PySynthMRI software version 1.0.0 with the source code and documentation is released on GitHub under a free GPL software license and can be accessed at: https://github.com/FiRMLAB-Pisa/pySynthMRI.git (accessed on 10 September 2023).

**Figure 2:**
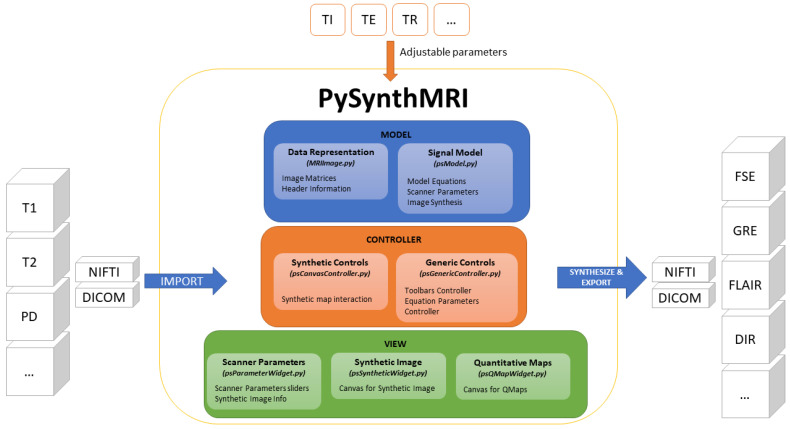
PySynthMRI architecture. The architecture follows the Model View Controller design pattern. PySynthMRI supports both DICOM and NIfTI files.

A scheme of its architecture is shown in Figure 2. The specific features of the tool are described below.

### 2.1. Synthetic Image Generation

The core functionality of PySynthMRI implements the generation of contrast images.

The tool interface provides a simple way to modify sequence parameters in order to choose values that generate the requested synthetic image, which is immediately shown to the user using the preferred interpolation for best rendering. The image synthesis is performed by calculating, pixel by pixel, the expected signal intensity as a function of a set of input images (for example, quantitative maps of T1, T2 and proton density) and simulated scanner parameters. The generic signal model that takes into account the effects of inversion recovery, T1 and T2 weighting is:

(1) $$S=abs\left(PD*\left(1-2*exp\left(-TI/T1\right)\right)*\left(1-exp\left(-TR/T1\right)\right)*exp\left(-TE/T2\right)\right)$$

PySynthMRI is distributed with a pre-defined set of sample presets for the generation of synthetic T1-weighted gradient-recalled echo (GRE), bias-free T1-weighted MPRAGE [14]/MP2RAGE [15], T2-weighted spin echo (SE), FLuid Attenuated Inversion Recovery (FLAIR) [16], Tissue Border Enhancement [17] and Double Inversion Recovery (DIR) [18]. The sample presets, as well as a sample input dataset consisting of quantitative maps of the T1, T2 and proton density of a healthy subject, are meant as a starting point to help the user get acquainted with the software. However, it is worth highlighting that PySynthMRI is designed to enable the full customization of the signal equations, which can be easily expanded to include additional input data, such as quantitative maps of T2* and magnetic susceptibility.

### 2.2. Data Formats

PySynthMRI provides complete support for the Digital COmmunications in Medicine standard (DICOM) and Neuroimaging Informatics Technology Initiative standard (NIfTI). Both standards can be used for importing the input data and exporting the synthetic images. Although NIfTI images include an extended header to store, amongst others, DICOM tags and attributes, they usually do not contain all tags requested by DICOM viewers [19]. To overcome this issue, PySynthMRI generates DICOM images using a template containing all necessary tags and automatically updates modified parameters, such as the TR, TE, TI, window scale, and series description. The Nibabel python library was used to handle both DICOM and NIfTI formats.

To achieve the proposed data-agnostic model, PySynthMRI maps the file format internally using an ad hoc Python data loader. The design choice allows developers to easily add new data formats if necessary.

### 2.3. Configuration File

**Figure 3:**
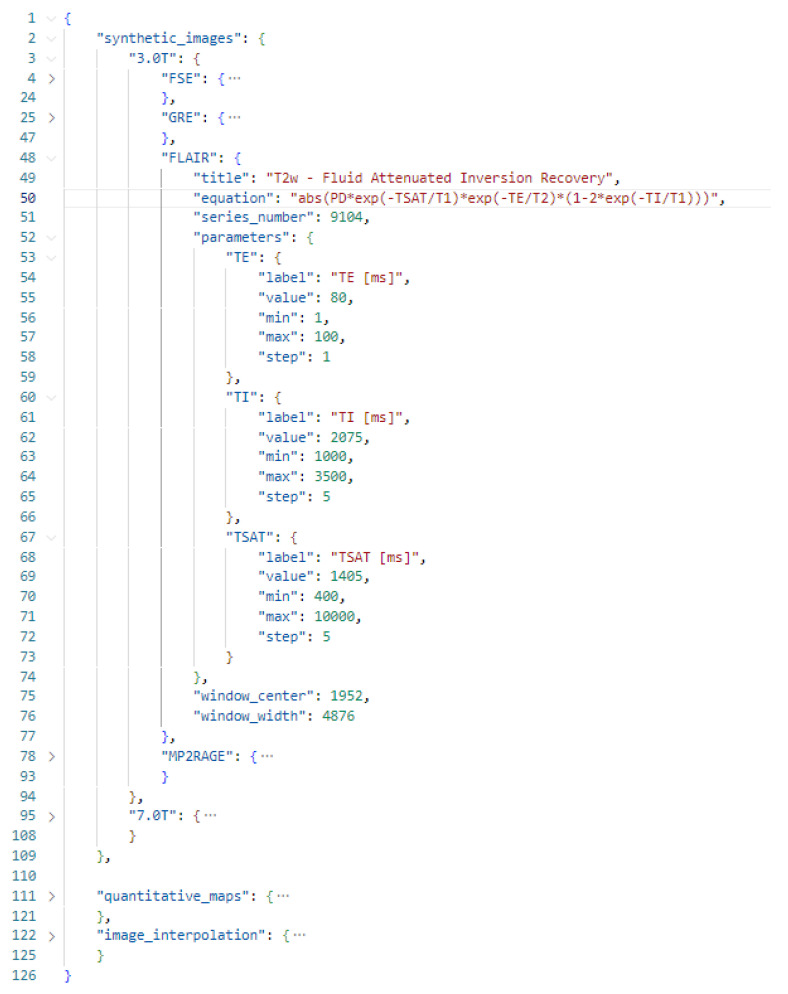
Example configuration file of PysynthMRI.

Signal models can be easily defined using a configuration file formatted as a JavaScript Object Notation (JSON) open standard format. The JSON format supports all data types needed by PySynthMRI, and it provides human readability, which is a key feature for easy access. An example of a configuration file is shown in Figure 3. Signal models of synthetic images are grouped in presets in order to facilitate the use of the appropriate set of signal models and parameters depending on the input dataset (for example, to load appropriate simulated scanning parameters for MR scanners operating at different fields). Each signal model is provided as a JSON object consisting of an equation that represents the actual model and the list of scanner parameters, along with their default values. The Configuration File contains the list of quantitative maps that are accepted by PySynthMRI, and it can be adjusted as desired and modified while using the tool: the updates are immediately applied. A validator checks for errors and inconsistencies each time the file is loaded, helping the user with the correct compilation. 

### 2.4. Graphical Interface Interaction

The interface of PySynthMRI is shown in Figure 1. The user can load the desired number of input images, which can be quantitative maps of T1, T2 and PD, as well as other types of maps (including user-defined binary masks). The files can be uploaded so as to provide a path or using the drag-n-drop feature.

The user can immediately evaluate the synthesized images resulting from simulated scanner parameters, which can be modified using sliders and/or mouse/keyboard interactions. The provided image viewer features include zoom, span and window greyscale regulation, as well as slicing and reformatting in the three orthogonal planes. Custom signal models can be added at runtime or using the Configuration File in order to obtain new synthetic contrasts.

### 2.5. Batch Synthesization

Although PySynthMRI is mainly intended for a direct graphical usage, one secondary feature is the execution of batch synthesization. The batch synthesization consists of automatically generating multiple contrast images from the provided input data of different subjects. The user selects the contrasts to be generated and provides the path containing the input data of different subjects. The tool sequentially synthesizes all the images without the need to export them individually. Batch synthesization drastically reduces the time required to apply synthetic MRI models to multiple acquisitions once the simulated acquisition parameters have been determined. 

### 2.6. Testing 

PySynthMRI has been tested by using input images representing maps of T1, T2 and PD obtained with different methods [10,20,21] on different scanners operating at 1.5T, 3T and 7T.

For demonstration purposes, a neuroradiologist with little programming skills and nine years of research experience used PySynthMRI to generate six types of synthetic images (T1w GRE and MP2RAGE [15]; T2w FSE and FLAIR [16]; TBE [17]; DIR [18]) from a set of input images consisting of maps of T1, T2 and PD obtained on a 3T scanner (MR750, GE Healthcare, Chicago, IL, USA). The input dataset was obtained using a 3D QTI sequence [10,20], consisting of an inversion-prepared steady-state free precession acquisition with spiral k-space sampling achieving the simultaneous quantification of PD, T1 and T2 of the whole head at a 1.1 mm isotropic resolution in 7 min. The user empirically edited the sample equations and the simulated scanning parameters to optimize the desired synthetic contrasts.

## 3. Results and Discussion

**Figure 4:**
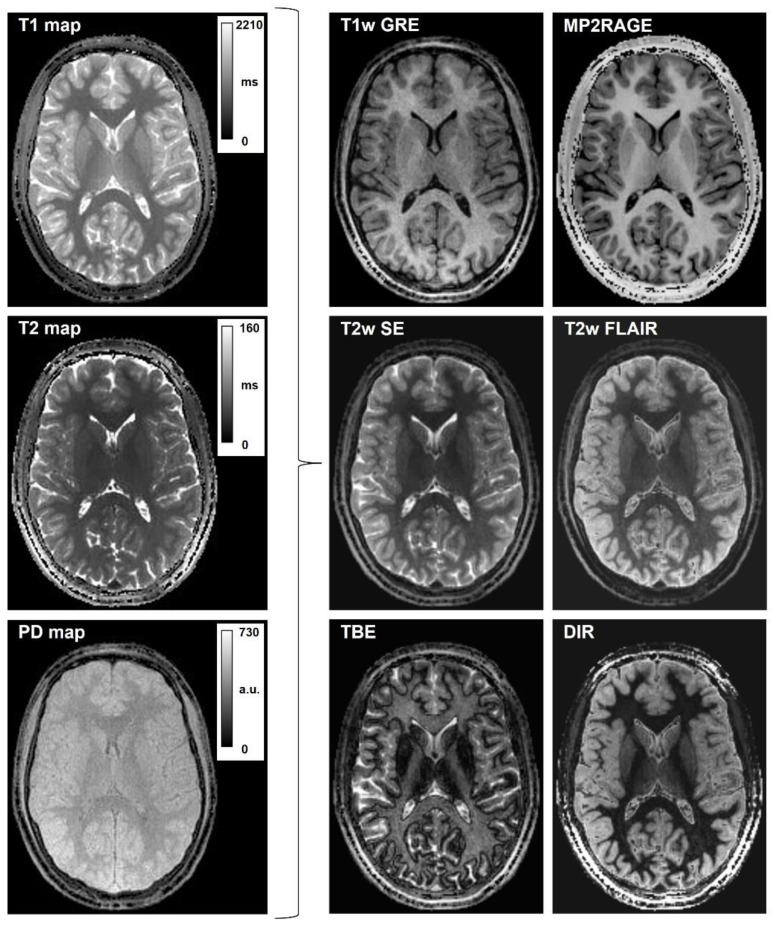
Synthetic T1-weighted and T2-weighted contrasts generated with PySynthMRI. On the left, the input quantitative maps; on the right, the synthesized images.

Figure 4 displays six types of synthetic images (T1w GRE and MP2RAGE; T2w FSE and FLAIR; DIR; TBE) obtained with PySynthMRI using maps of T1, T2 and PD of one subject (18-year-old male) as input. To obtain these images, the user empirically edited the sample equations and set the simulated scanning parameters.

- Synthetic T1-weighted images simulating GRE (Gradient-recalled Echo) were synthesized with the following equation, incorporating the input maps of PD and T1 and setting the simulated acquisition parameter TR:
  (2) $$\begin{array}{c} S_{GRE}=PD*\left(1-exp\left(-TR/T1\right)\right)\\ \mathrm{TR}\;=\;113\;\mathrm{ms}. \end{array}$$
- Bias-free T1-weighted MPRAGE was synthesized by taking into consideration that B1 bias in QTI is incorporated in the PD map, which can be excluded from the formula, achieving a similar appearance to MP2RAGE uniform imaging [15]:
  (3) $$\begin{array}{c} S_{MP2RAGE}=abs\left(1-2*exp\left(-TI/T1\right)\right)\\ \mathrm{TI}\;=\;1816\;\mathrm{ms} \end{array}$$
- Synthetic T2-weighted images simulating a SE (Spin Echo) acquisition were obtained as follows:
  (4) $$\begin{array}{c} S_{SE}=PD*\left(1-exp\left(-TR/T1\right)\right)*exp\left(-TE/T2\right)\\ \mathrm{TR}\;=\;8000\;\mathrm{ms};\;\mathrm{TE}\;=\;80\;\mathrm{ms} \end{array}$$
- Synthetic T2-FLAIR was obtained with a user-modified formula incorporating a coefficient TSAT, which enables the introduction of T1-weighting, which better mimics the one in conventional imaging:
  (5) $$\begin{array}{c} S_{FLAIR}=abs\left(PD*exp\left(-TE/T2\right)*exp\left(-TSAT/T1\right)*\left(1-2*exp\left(-TI/T1\right)\right)\right)\\ \mathrm{TE}\;=\;80\;\mathrm{ms};\;\mathrm{TSAT}\;=\;1405\;\mathrm{ms};\;\mathrm{TI}\;=\;2075\;\mathrm{ms}. \end{array}$$
- Synthetic TBE acquisitions were obtained from the generic signal model, with the appropriate parameters TI, TR, TE:
  (6) $$\begin{array}{c} S_{TBE}=abs\left(PD*\left(1-2*exp\left(-TI/T1\right)\right)*\left(1-exp\left(-TR/T1\right)\right)*exp\left(-TE/T2\right)\right)\\ \mathrm{TR}\;=\;5020\;\mathrm{ms};\;\mathrm{TE}\;=\;1\;\mathrm{ms};\;\mathrm{TI}\;=\;795\;\mathrm{ms} \end{array}$$
- Double Inversion Recovery (DIR [18,22]) was obtained by including the two times of the inversion TI1 and TI2 in the signal equation, as follows:
  (7) $$\begin{array}{c} S_{DIR}=abs\left(PD*\left(1-2*exp\left(-TI2/T1\right)+2*exp\left(-\left(TI1+TI2\right)/T1\right)-exp\left(-TR/T1\right)\right)*exp\left(-TE/T2\right)\right)\\ \mathrm{TR}\;=\;6670\;\mathrm{ms};\;\mathrm{TE}\;=\;80\;\mathrm{ms};\;\mathrm{TI}1\;=\;2208\;\mathrm{ms};\;\mathrm{TI}2\;=\;545\;\mathrm{ms} \end{array}$$

The user was able to obtain the desired synthetic images without any programming skills.

**Figure 5:**
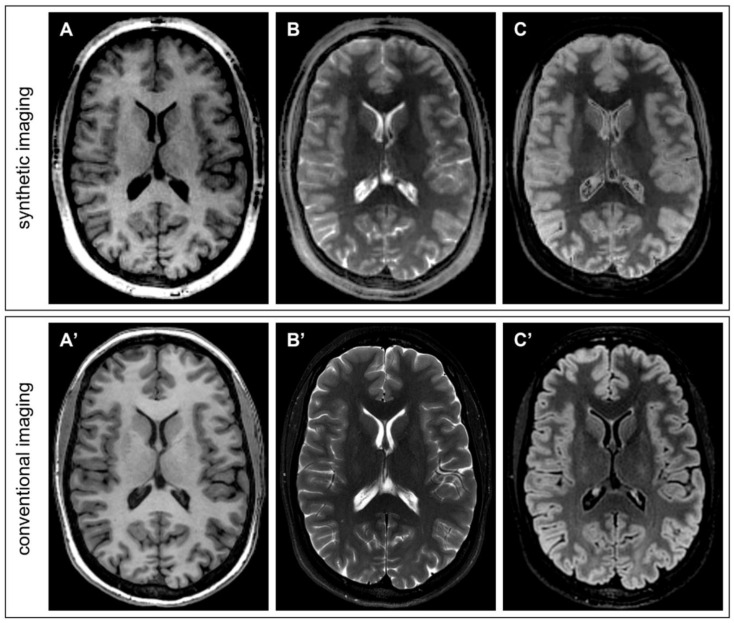
Direct qualitative comparison between synthetic images obtained with PySynthMRI from QTI-derived maps (**top row**) and conventional T1-weighted (**A**,**A’**), T2-weighted (**B**,**B’**) and T2-FLAIR (**C**,**C’**) images of conventional acquisitions (**bottom row**).

For a qualitative comparison with images obtained with conventional acquisitions, Figure 5 displays three synthetic image contrasts generated with PySynthMRI (T1-w, T2w and T2-FLAIR) using the quantitative maps obtained with one single QTI acquisition (total acquisition time: 7 min) from a 28-year-old female, compared with the images obtained with conventional 3D FSPGR (TR = 8.1 ms; TE = 3.2 ms; TI = 450 ms; spatial resolution = 1 × 1 × 1 mm^3^; scan duration = 4′11″), 2D FSE (TR = 3636 ms; effective TE = 113.3 ms; resolution = 0.57 × 0.57 × 4 mm^3^; scan duration = 2′11″) and 3D T2-FLAIR (TR = 7000 ms; effective TE = 113.3 ms; TI = 1945 ms; resolution = 1.16 × 1.16 × 1.2 mm^3^; scan duration = 5′25″).

The storage size is exactly the same for synthetic and conventional images with the same resolution and coverage.

Synthetic FLAIR image (Figure 5C) fails in fluid suppression and artifacts are visible in the basal ganglia. Those artefacts are not related to PySynthMRI but are instead related to the input maps that were used for testing the software. QTI acquisition is known to provide erroneous relaxation estimates in moving tissue, that is, in particular, in the ventricular cerebrospinal fluid. As previously reported in the literature, the FLAIR artefacts are also caused by partial volume effects at the interface between periventricular tissue and cerebrospinal fluid [23]. One possible way of mitigating the problem of partial voluming is to leverage the software so as to include an additional input map in the signal model (e.g., a water fraction map) that can be produced from the same QTI acquisition used for T1 and T2 mapping. Furthermore, the synthesized FLAIR can be improved using a deep-learning method based on the Generative Adversarial Network [24].

The synthetic images were saved in DICOM and NIfTI formats, suitable for radiological evaluation and storage on conventional PACS as well as for further image processing/analysis using external software. The same presets were applied to a batch of 210 different patients (including patients with neurodegenerative diseases, neurodevelopmental disorders, epilepsy, and brain tumors) in order to provide six radiological contrasts for each patient, resulting in the generation of 210 × 6 = 1260 3D volume images, which were saved in the DICOM format. The processing time was 1.1 s per 3D synthetic image on a Windows machine equipped with an i5-8500 CPU @ 3.0 GHz, 6 cores and 16 GB RAM. 

Despite the fact that we tested PySynthMRI on the provided signal models, it is possible to include further input maps, such as B1 field and/or noise models, which may, on the one hand, have limited use in the generation of synthetic images for radiological inspection but may, on the other hand, have a potential use when the software is used for educational purposes.

The development and dissemination of time-efficient quantitative MRI techniques motivate the use of PySynthMRI in clinical research. It allows specialists to easily synthesize, at post-processing time, multiple contrasts for a particular subject. This function can be useful for refining the desired contrasts, as optimal parameters depend on the patient age, region of interest and/or pathology. The synthesization process can be useful in cases where it is desirable to generate contrasts not originally considered at the time of acquisition. 

PySynthMRI also shows a high potential in education, as it can be used to show how sequence parameters impact the generation of images with different tissue contrasts.

PySynthMRI complements other types of software whose main purpose is the accurate simulation of the MR signal. In this respect, it is worth referring to simulation tools such as SIMRI [25], JEMRIS [26], MRiLab [27] and others [28,29,30]. These simulators are primarily intended as tools for MRI physicists to support the development of new sequences and the optimization of acquisition parameters. On the contrary, the primary function of PySynthMRI is that of an interactive viewer tailored for radiologists in a clinical research context, and it is aimed at evaluating and producing synthetic contrasts in real time. Its computation time for one entire 3D volume is in the order of 1 s, while for signal simulators, it is in the order of minutes or hours. The MRI community may take advantage of the future development of tools in which both of these complementary aspects (that is, an accurate MRI signal simulation and the production of synthetic images for radiological purposes in a clinical context) are implemented.

The modular architecture allows developers and contributors to easily extend and customize the tool to incorporate additional parameter maps, such as T2* or MT, or different predefined contrast images. Future releases of PySynthMRI will aim to incorporate further capabilities, including an automatic tissue segmentation software module capable of computing and showing probabilistic maps of white matter (WM), gray matter (GM) and cerebrospinal fluid (CSF) using a pre-built lookup table representing the relationship between partial volume tissue composition and provided quantitative maps [31].

## Data Availability

Software and sample data are available at https://github.com/FiRMLAB-Pisa/pySynthMRI.git (access date: 10 September 2023).
